# Nuclear-Based Labeling of Cellular Immunotherapies: A Simple Protocol for Preclinical Use

**DOI:** 10.1007/s11307-024-01923-z

**Published:** 2024-07-03

**Authors:** Alessia Volpe, Serge K. Lyashchenko, Vladimir Ponomarev

**Affiliations:** 1 Department of Radiology, Memorial Sloan Kettering Cancer Center, 1275 York Avenue, New York, NY 10065 USA; 2 Center for Cell Engineering, Memorial Sloan Kettering Cancer Center, 1275 York Avenue, New York, NY 10065 USA; 3 Molecular Pharmacology and Chemistry Program, Memorial Sloan Kettering Cancer Center, 1275 York Avenue, New York, NY 10065 USA

**Keywords:** Nuclear imaging, Preclinical imaging, Direct labeling, Reporter gene labeling, Cell therapy

## Abstract

Labeling and tracking existing and emerging cell-based immunotherapies using nuclear imaging is widely used to guide the preclinical phases of development and testing of existing and new emerging off-the-shelf cell-based immunotherapies. In fact, advancing our knowledge about their mechanism of action and limitations could provide preclinical support and justification for moving towards clinical experimentation of newly generated products and expedite their approval by the Food and Drug Administration (FDA).

Here we provide the reader with a ready to use protocol describing the labeling methodologies and practical procedures to render different candidate cell therapies *in vivo* traceable by nuclear-based imaging. The protocol includes sufficient practical details to aid researchers at all career stages and from different fields in familiarizing with the described concepts and incorporating them into their work.

## Introduction

Cellular immunotherapies comprise a wide and constantly expanding arsenal of different immune cell therapeutics [1]. As such, they are based on living cells, either autologous (isolated from the same patient) or allogeneic (isolated from a different donor), whose role in the successful treatment of a broad range of human diseases has consolidated over the years. New emerging off-the-shelf cell-based immunotherapies are being developed worldwide by scientists and are conventionally tested in preclinical animal models without a reliable assessment of their *in vivo* distribution, targeting capability and long-term survival. Molecular imaging of immune cell therapeutics could provide unique insights into their mechanism of action, their success and, sometimes, their failure [2]. To render them traceable, either molecular probes or contrast-generating reporters must be introduced into the cells by employing direct or indirect labeling methodologies [3, 4]. When doing so, aspects such as preclinical *versus* clinical setting, tracking time, tracking intervals, labeling strategy, modality, and probe of choice, need to be carefully considered [5]. Unlike other molecular imaging modalities, preclinical nuclear-based imaging combines non-invasive whole-body tracking capabilities with exquisite sensitivity and depth penetration [6], enhanced resolution (with Positron Emission Tomography (PET) offering < 1mm resolution) [7, 8], multiplexing capabilities [9], and absolute 3D quantification.

**Fig. 1:**
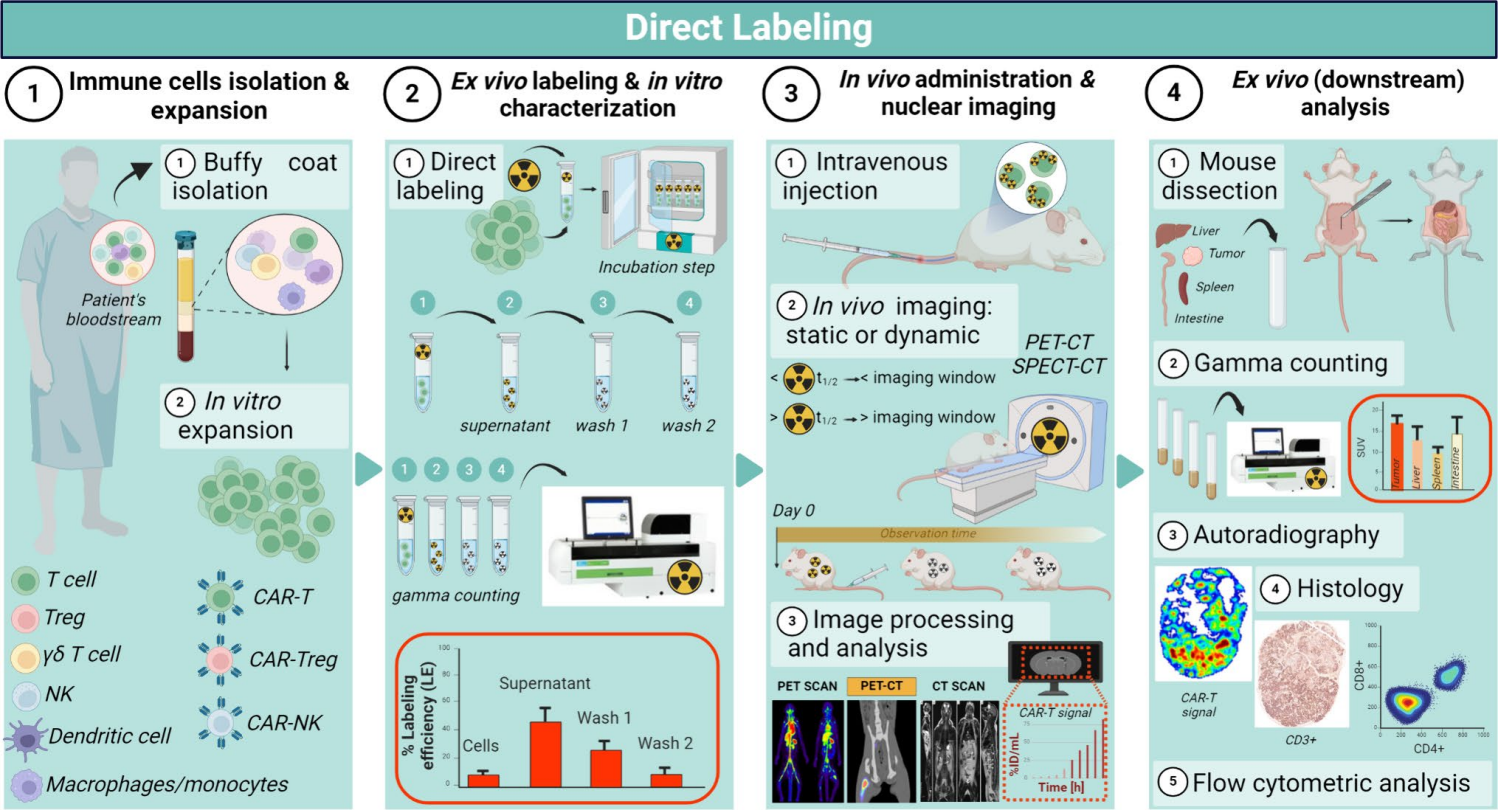
Schematic representation of the direct labelling experimental workflow

**Fig. 2:**
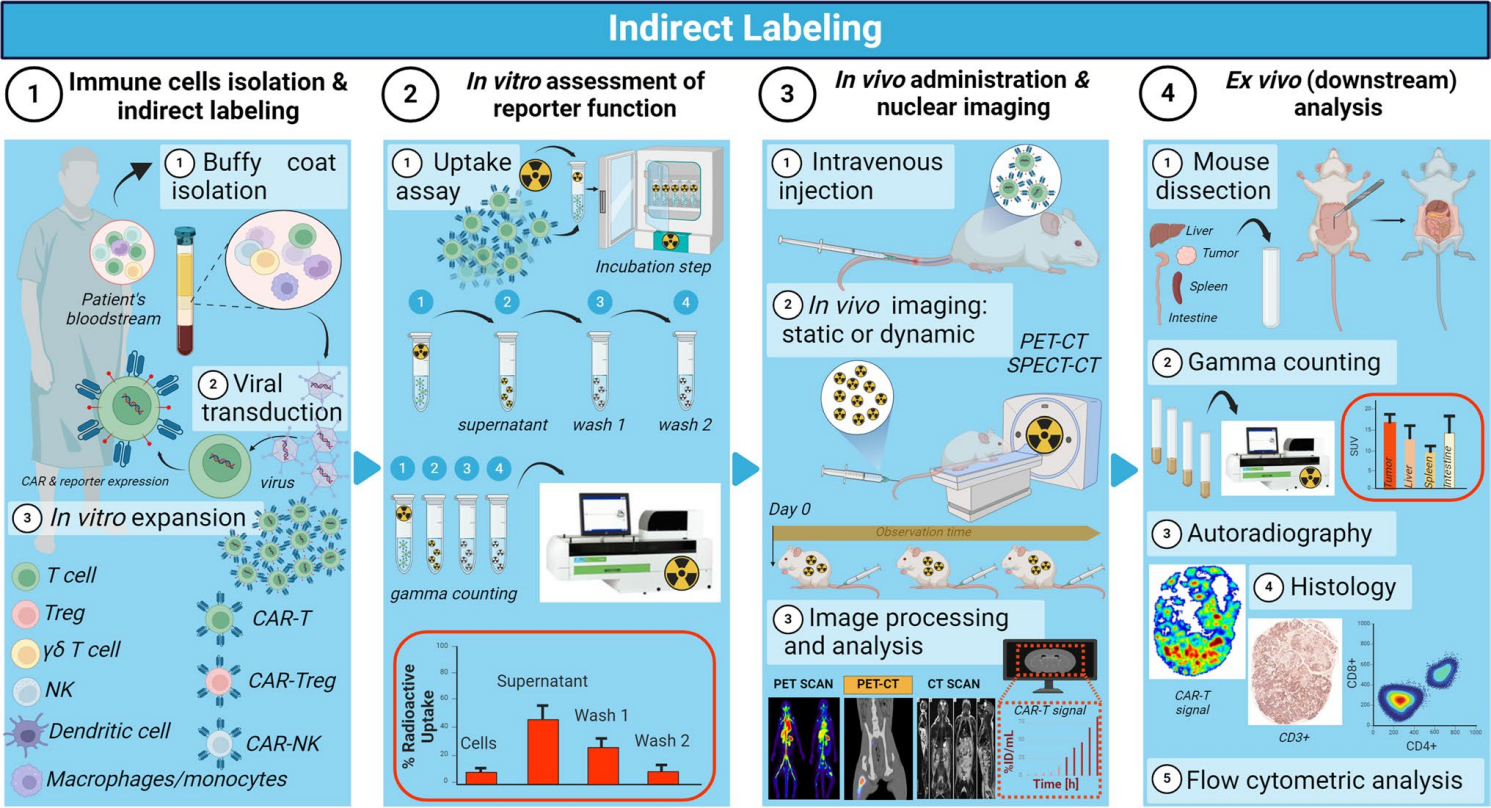
Schematic representation of the indirect labelling experimental workflow

Hereby we describe a simple and comprehensive protocol for the nuclear-based labeling of immune cell therapeutics and allowing the reliable assessment of their *in vivo* fate and therapeutic efficacy by whole-body non-invasive preclinical imaging. The following direct and indirect methods and respective experimental workflows (Figs. 1 and 2) are tailored to the visualization and monitoring of different classes of cell-based therapies, including adoptively transferred T (Chimeric Antigen Receptor T) cells [10, 11], natural killer (NK) [12, 13] and CAR-NK cells [14], regulatory T cells (Treg) [15] and CAR-Tregs [16, 17], gamma delta T cells [18, 19] and dendritic cell vaccines [20]. Detailed downstream *in vitro*, *in vivo* and *ex vivo* validation experiments are also included. A list of Materials was also included at the end of the protocol. Whenever applicable, we provide the reader with critical considerations and suggestion for the successful execution of this protocol. The application of these concepts should exclusively involve fully trained personnel.

## Experimental Protocol for Preclinical Use

CAUTION: As this protocol involves handling and manipulation of radioactive material and or animals, researchers should consult with their home institution’s Radiation Safety and /or Medical Physics Department and undergo the required training prior to initiating this work. Steps should be taken to minimize exposure to ionizing radiations, including wearing whole-body and ring dosimeters. Gloves should be worn at all times. Radioactive waste MUST be disposed according to institutional radiation safety and local waste management guidelines. Existing home institution SOPs (Standard Operating Procedures) MUST be strictly followed for your own safety and that of your coworkers.

Cyclotrons, generator systems, linear accelerators (LINACs) or nuclear reactors can be used as a source of radioactive isotopes for this purpose. In either of these cases, access to a radiochemistry facility is essential. Radioisotopes and radioactive probes suitable to label immune cells need to be produced with high radiochemical purity (RCP; general consensus is ≥95%, however tracer dependent) and yield (RCY; tracer dependent). To achieve adequate radio-incorporation of the radionuclide into the targeting vector molecules, several radionuclide precursor solution considerations must be taken into account, including the radionuclide specific activity as the activity per unit mass of a radionuclide (radioactivity/mg) [21], possible trace contamination with elemental impurities that may compete for radio-incorporation with the radionuclide of interest, and the chemical form of both radionuclide itself and its formulation. In most instances, this information can be obtained by reviewing the Certificate of Analysis (CoA) that has been provided by the radionuclide manufacturer along with the material. Also, because the chemical form of the radionuclide solution may change over time, as a general rule, the time between radiochemical isolation of the radionuclide by the radionuclide producer and radiolabeling should be minimized. For more detailed radioisotope considerations and radiochemical probe design, refer to Volpe et al. [22].

Cells isolation protocols and culture medium may vary depending on immune cell type (T lymphocytes [23], NK (natural killer) cells [24], Tregs [25, 26] (regulatory T cells), gamma delta T cells [27], and dendritic cells [28]. Some cell types can be isolated using more than one protocol (see Hoerster *et al.* and Sato *et al.*) [24, 29]. For further details on different isolation protocols, practical isolation steps, required reagents, materials, and equipment, we refer the reader to commercially available kits.

Prior to labeling, cells are *in vitro* expanded, counted, and washed with phosphate buffer saline (PBS; Ca^2+^/Mg^2-^ free) before being resuspended in PBS at room temperature (RT) (Note 1^1^; Note 2^2^)

1. *Direct (ex vivo) Labeling*
  1.1. Add to 1-10x10^6^ cells the desired radioisotope of choice (Note 3^3^; Note 4^4^; Note 5^5^). To obtain optimal labeling, preliminary optimization and calculation of incorporation rates are required.
  1.2. Incubate resuspended cells for 10-30 min at 37 degrees Celsius and 5% CO_2_ on agitating rack (at 350 rpm) (Note 6^6^). Incubation time may vary depending on cell type and the probe.
  1.3. Wash cells three times in ice-cold PBS (not containing Ca^2+^/Mg^2-^). Centrifuge and collect each wash solution in three separate collection tubes labeled “supernatant”, (wash 1” and “wash 2”, respectively.
  1.4. Cells are resuspended in growth medium or PBS for further *in vitro* and/or *in vivo* experiments.
  1.5. Radioactivity in resuspended cells and respective washes is measured using a gamma-counter measuring the radioisotope of choice (Note 7^7^).
  1.6. Radioactive cell-labeling efficiency is calculated using Equation 1.

1 $$LE\ \left(\%\right)=\left(\frac{activity\ of\ cell\ fraction}{activity\ of\ cell\ fraction+ activity\ of\ combined\ washes}\right)x100$$

1.7. To evaluate the long-term radiotracer retention, radiolabeled cells are cultured at a confluence of 0.5x10^6^ cells/mL medium. Samples are centrifuged at 1500 rpm and supernatants are removed and collected in prelabeled tubes. Cell pellets are re-suspended in their culture medium. Cell-associated radiotracer and supernatants are measured at the gamma counter and percentages are calculated using Equation 1. As cells continue to divide, expect a label/signal dilution over-time as the label will redistribute to the daughter cells. The percentage of total activity bound to the cells will decrease with time compared to the initial measurement according to radiotracer decay (the latter depending on half-life of the chose radiotracer), but cell expansion will not be detectable as the number of individually labeled cells will decrease [3], making the latter method unsuitable for a reliable long-term observation of fast-dividing cells.

IMPORTANT: Any commercially available gamma counter will provide decay corrected counts. In the unlikely circumstance that is not the case, a sample of known activity from the initial radioactive stock can be re-measured alongside with experimental samples. In lieu of these options, radioactivity at any given time can be calculated using the standard formula:

2 $${A}_t={A}_0\cdot {e}^{-\lambda \cdot t}$$

Or its derivatization:

3 $${A}_0={A}_t\cdot {e}^{-\lambda \cdot -t}$$

where A is the activity, A_0_ is the initial activity, A_t_ is the activity after any time t, and λ is the decay constant, which for most radioactive material is readily available through the literature, or can be otherwise calculated using the following formula:

4 $$\lambda =\frac{\ln 2}{t_{1/2}}$$

IMPORTANT: Not all radioactive probes are well suited candidates for the direct labeling of cell-based immunotherapies. One example is provided by Jacob *et al.*, reporting on dose-dependent changes in polyclonally expanded human Tregs phenotype (downmodulation of CD4 and CD25), impaired proliferation both *in vitro* and *in vivo*, and failure to survive when labeled with ^89^Zr-oxine [31]. Always perform downstream assays to assess the consequences of direct radiolabeling, including viability, phenotyping, proliferation, functional and DNA damage studies. Also consider using a different PET radiotracer with a similar half-life (days) but with a potentially less harmful radioactive decay than ^89^Zr for this specific immune cell type.

2. *Indirect (reporter gene-based) Labeling Method:*
  2.1. Viral Particles Generation
    2.1.1. Viral constructs (Note 8^8^) are generated bearing the following components: (i) a “regulatory complex” (Note 9^9^); (ii) a “reporter gene”; (iii) a targeting receptor (*e.g.*, CAR); (iv) a selection marker (*e.g.*, either a fluorescent protein or an antibiotic resistance)
    2.1.2. Either highly efficient viral-producing cells (*i.e.*, 293^RD^ [39, 40], and Phoenix Ampho) [41] or stable cells clonally selected for higher titer (*i.e.*, PG13) [42] can be purchased through the American Type Culture Collection (ATCC) (Note 10^10^)
    2.1.3. The day prior transfection, seed cells in either 6-well plate or 10 cm plate to achieve 50-70% confluence on the next day (gently shake the plate to avoid uneven distribution) (Note 11^11^)
    2.1.4. On the next day, proceed with preparing the transfection mix by adding the required plasmid DNA and the transfection reagent (*e.g.*, X-tremeGENE™, Lipofectamine™, Polyethyleneimine (PEI)). Always maintain sterile conditions
    2.1.5. Incubate the transfection mix for 10 min at RT
    2.1.6. Replace the growth medium of the virus producing cells with fresh one (without L-glutamine or antibiotics)
    2.1.7. Gently add the transfection mix to the cells without disturbing them
    2.1.8. Incubate plates at 37 °C and 5% CO_2_. Then, inspect the plates using a tissue culture microscope and gently replace the media with fresh medium (without L-glutamine or antibiotics)
    2.1.9. 48h later, the viral supernatant is ready to be harvested (please follow step 2.2.3 onwards)

2.2. Cellular immunotherapies transduction

This is a general protocol for immune cells transduction. Immune cells are non-adherent cells and therefore transduction protocols will require the use of non-tissue culture treated plates and pre-coating with RetroNectin. Transduction is performed on a 6-well format with 1 x 10^6^ cells per well but both plate format and number of cells can be scaled up or down if needed.

- 2.2.1 Pre-coat a culture plate with RetroNectin (add at least 1mL per well in a 6-well plate to ensure complete surface coverage; scale quantities up or down depending on the chosen plate format)
- 2.2.2 Preserve plate sterility and prevent RetroNectin evaporation by sealing it with parafilm. Then store overnight at 4 °C or for 2h at RT
- 2.2.3 Harvest viral containing supernatant from virus producing cells and filter them through a 0.45 μm syringe filter (Note 12^12^)
- 2.2.4 Replace media in the virus producing cells for subsequent transduction rounds
- 2.2.5 Supplement viral containing media with interleukin-2 (IL-2). Final concentrations used may vary depending on immune cell type (*e.g.*, for T cells, 20 IU/mL; for Tregs 1000 IU/mL; for gamma delta T cells 300-1000 IU/mL) (Note 13^13^)
- 2.2.6 Remove RetroNectin coating by aspiration and wash wells with 1 mL PBS supplemented with 10% FCS and leave at RT for 30 min
- 2.2.7 Collect immune cells (Note 14^14^) and proceed counting them with using either an hematocytometer or an automated cell counter
- 2.2.8 Take the desired cell number for transduction and centrifuge at 500 x *g* for 5 min at 4 °C
- 2.2.9 Resuspend pellet in filtered viral containing supernatant at 1 x 10^6^ cells/3 mL and incubate for 15 min at RT (Note 15^15^)
- 2.2.10 Remove PBS (without Ca^2+^/Mg^2+^) with 10% FCS by aspiration and wash once with regular PBS (without Ca^2+^/Mg^2+^)
- 2.2.11 Aliquot 3 mL of supernatant containing 1 x 10^6^ cells per well.
- 2.2.12 Perform spinoculation by centrifuging the plates at 100 x *g* for 1 h at 4 °C (Note 16^16^)
- 2.2.13 Incubate plates at 37 °C and 5% CO_2_
- 2.2.14 Repeat transduction after 4-6 h. Repeat again on the next day for a total of four transductions (Note 17^17^)
- 2.2.15 Before repeating the transduction, centrifuge the plate at 500 x *g* for 5 min
  - Repeat steps 2.2.3 to 2.2.5
  - Remove about 2 mL of old viral supernatant by aspiration and add 3 mL fresh filtered one
  - Add fresh filtered viral supernatant to the cells and continue from step 2.2.12
- 2.2.16 48h after the last transduction round, spin plates at 500 x *g* for 5 min
- 2.2.17 Gently aspirate supernatant containing viral particles
- 2.2.18 Wash cells twice in PBS (without Ca^2+^/Mg^2+^)
- 2.2.19 Resuspend cells in their fresh culture medium supplemented with IL-15 (Note 13), move into a larger flask and keep in the incubator at 37 °C and 5% CO_2_

To ensure high viral titer and subsequent efficient transduction of immune cells, we recommend optimizing the Multiplicity of Infection (MOI; representing the number of viral particles required to achieve a 100% transduction). To calculate the MOI, use the following Equation 5:.

5 $$MOI=\frac{number\ of\ viral\ particles}{number\ of\ target\ cells}$$

2.3. *In vitro* characterization of transduced cellular immunotherapies:
  2.3.1. *In vitro* assessment of successful transduction can be done by flow cytometric analysis if the reporter bearing construct also includes a fluorescence marker (*e.g.*, if reporter is co-expressed with Green Fluorescent Protein (GFP), it can be detected by flow cytometry). For accurate quantification of reporter expression, we recommend performing quantitative flow cytometry (QFCM). If expression is too low (<95%), select positive clones by fluorescence-activated cell sorting (FACS). If the reporter-based vector also includes an antibiotic resistant gene, the corresponding antibiotic is used as a selection marker in the cultured media to maintain the population pure (>99% reporter+). Remember to use an appropriate negative control (parental non-transduced cells) to determine flow cytometer settings. If/when available, we suggest to also use a positive control (*e.g.*, a similar cell type previously transduced with the same transgene, thus offering expression of the same FP (fluorescent protein)
  2.3.2. *In vitro* assessment of reporter function implies performing a radioactive uptake assay. The type of radioisotope varies depending on the chosen reporter gene [5]
    2.3.2.1. Aliquot 1 x 10^6^ cells in pre-labeled Eppendorf tubes called “cells”. Prepare samples in triplicates and include also “control samples” corresponding to non-transduced parental cells (Note 18^18^)
    2.3.2.2. Wash cells twice with ice-cold PBS
    2.3.2.3. Incubate cells with desired amount of radioactivity (suggested is 1.35-1.50 μCi per sample) in 1 mL PBS for 30 min-1 h at 37 °C and 5% CO_2_ (Note 2)
    2.3.2.4. Blocking or competitive studies as “specificity controls” can be performed depending on the reporter (*e.g.*, sodium perchlorate is used as a competitive substrate for the sodium iodide symporter NIS and resulted in uptake depletion demonstrating radioactive uptake specificity) [48]. In that case, pre-treat cells with blocking or competitive agent, then add it again with the radioactivity (keep concentration constant throughout the assay). We encourage to also add a negative control (parental non-transduced cells) to determine basal uptake levels
    2.3.2.5. Centrifuge samples at 1500 rpm at RT, collect 100 μL of supernatant and transfer it into a pre-labeled tube named “supernatant”. Safely remove the remaining 900 μL and dispose of it according to institutional radiation safety guidelines
    2.3.2.6. Wash the cells twice with ice-cold PBS alternating centrifugation steps. Collect 100 μL from each washing solution and transfer them into pre-labeled tubes named “wash 1” and “wash 2”, respectively
    2.3.2.7. Follow steps 1.4 to 1.5.
    2.3.2.8. Calculate radiotracer uptake using the following Equation 6, where CPM represents the decay-corrected radioactivity counts per minute (Note 19^19^)

6 $$\% Uptake=\left(\frac{CPM(cells)}{CPM\ (cells)+ counts\ of\ combined\ washes\ \left( supernatant+ wash\ 1+ wash\ 2\right)}\right)\ x\ 100$$

New emerging cell therapeutics (including human cardiac progenitor cells and human-induced pluripotent stem cells (iPSCs)) are also amenable to indirect labeling, and step-by-step protocols describing their engineering using the sodium iodide symporter NIS reporter gene are available [49, 50].

IMPORTANT: When the chosen reporter gene is a transporter or a surface protein, localization studies must be performed (confocal microscopy) prior to the radioactive uptake assay (as described in 2.3.2) to prove its correct localization as a fundamental pre-requisite for its correct function (imaging capability shown by the successful uptake of desired radioactive probe). Staining controls (*e.g.*, membrane marker) and negative controls (parental non-transduced cells) are required (see Volpe et al. as an example) [47].

IMPORTANT: Whether Direct or Indirect Labeling was performed, we strongly recommend the reader to perform all application-dependent downstream experiments, including assessing the effects of cell labeling on their long-term viability, proliferation, phenotype, functional status (including activation markers and/or CAR expression), cytotoxic capability and potential radiation- induced DNA damage as compared to the parental cells (see referenced examples) [24, 31, 47]. These are crucial steps to determine whether the labeling procedure had detrimental effects on the cellular function and are a sole responsibility of each user. Perform these experiments alongside a negative control (parental non-transduced cells). Other controls may be needed depending on the type of experiment and its complexity and will therefore not be discussed in this protocol.

3. *In vivo tracking of cell-based immunotherapies in relevant preclinical models:*

IMPORTANT: Prior to animal testing, ensure that the *in vivo* protocol is approved by the local Ethical Review Panel and is compliant with the institutional ethical and safety guidelines and regulations. Laboratory personnel is required to undergo training before performing invasive procedures in living animals. Access to an imaging facility is essential to perform the following protocol.

Relevant therapeutic models will have to be established prior to starting the labeling of therapeutic cells. The choice of genetic backgrounds and animal strains (*i.e.*, humanized, fully immunocompetent or with various levels of immune compromisation) will depend on study goals and is a sole responsibility of the researcher (see Duncan *et al.* [51] for CAR-T cell models; for a general overview of available murine models for cancer immunotherapy, refer to Olson et al. [52]).

3.1. *Ex vivo* radiolabeled cells:
  3.1.1. Further to the washing (as described in step 1.3), centrifuge radiolabeled cells at 1500 rpm for 5 min at RT
  3.1.2. Remove radioactive supernatant without disturbing the cell pellet and safely dispose of it
  3.1.3. Resuspend cells in 50-200 μL saline or serum-free medium (Note 20^20^)
  3.1.4. Keep cells on ice until *in vivo* intravenous administration
  3.1.5. Aliquot a sample of radiolabeled cells and washes for further *in vitro* testing (*i.e.*, gamma counting and radiotracer retention; see steps 1.5 and 1.7 of 1.1, respectively)
  3.1.6. Prepare animals for injection. If interested in the distribution kinetics of directly labeled therapeutic cells once in the body, a dynamic scan will be required. Therefore, animals must be anesthetized beforehand (please follow steps described in Note 25) in an induction chamber with 1.5-2.0% (v/v) in O_2_ (flow rate 1-1.5 L/min) (Note 21^21^)
  3.1.7. Assess anesthetic depth using either the tail pinch or the pedal reflex method
  3.1.8. Place the mouse onto a heating pad to maintain physiological temperature and with its nose in an anesthetic supply mask and proceed with warming the tail using an infrared light lamp
  3.1.9. Slowly inject radiolabeled cells intravenously via tail vein catheter or using a syringe connected to a hypodermic needle (gauge 29-31) (Note 20 and Note 22^22^)
  3.1.10. If no dynamic imaging is needed post injection, perform intravenous injection of radiolabeled cells into conscious animals (skip steps 3.1.6 to 3.1.8). However, to this date, no published literature is available describing changes in the biodistribution of any cell-based therapeutic product upon administration under anesthesia.
  3.1.11. If imaging is needed post injection, place the mouse onto the bed of a microSPECT-CT or microPET-CT scanner and ensure that anesthetic supply is constant, and animal is asleep
  3.1.12. Ensure correct positioning of the animal (*e.g.*, sphinx-like position)
  3.1.13. Before starting the imaging, ensure that animal monitoring devices are installed (including an ECG pad to record the electrocardiogram and a rectal temperature probe for temperature monitoring)
3.2. Reporter gene labeled cells:
  3.2.1. Start with animal preparation (see steps 3.1.6 to 3.1.8 of “*Ex vivo* radiolabeled cells” subsection)
  3.2.2. Intravenously inject therapeutic cells expressing the desired radionuclide-based reporter into anesthetized mice (Note 22)
  3.2.3. If no imaging is needed post injection, perform intravenous injection of radiolabeled cells into conscious animals (skip steps 3.1.6 to 3.1.8)
  3.2.4. If imaging is needed post injection, inject the reporter-paired radioactive probe, and allow blood clearance prior to imaging (Note 23^23^)
  3.2.5. Follow steps 3.1.11 to 3.1.13
  3.2.6. To observe the early stages of cell immunotherapies distribution, inject the radioactive probe immediately after the cells. If prolonged monitoring is needed, the radioactive probe can be administered at the desired time point and repeated imaging can be performed indefinitely (Note 24^24^)

4. *Nuclear imaging by microSPECT-CT or microPET-CT and data analysis:*

Parameters such as *in vivo* distribution, metabolism and clearance from the body may vary depending on the radioactive probe used (refer to Volpe *et al.* for preferential distribution and properties of available radioactive probes) [22]. Therefore, it is important to add some waiting time prior to imaging in order to allow radiotracer *in vivo* biodistribution and avoid high blood signal.

Depending on the radiotracer characteristics (gamma rays or positron emitter), either SPECT (Single-photon emission computed tomography) or PET (Positron emission tomography) will be used.

4.1. Set the desired CT parameters (*e.g.*, 55 kVp tube voltage, 1200 ms exposure time with one-degree angular stepping, 360-degree projections). When ready, acquire CT image to provide anatomic information and accurate tissue attenuation correction of SPECT or PET
4.2. If performing SPECT imaging, set the parameters for image acquisition (*e.g.*, for ^99m^Tc[TcO_4_^-^], perform a 45 min scan, 40 frames, 360-degree projections, collimator pinhole size 1 mm, 110-150 keVp energy window). Then acquire SPECT image
4.3. If performing PET imaging, set the parameters accordingly (*e.g.*, for [^18^F]BF_4_^-^: perform a 30 min scan, 1:5 coincidence mode, 400-600 keVp energy window). Then acquire PET image (Note 25^25^ and Note 26^26^)
4.4. If repeated animal imaging is needed, allow animals to fully recover from anesthesia and transfer them into a maintenance unit
4.5. If this is a terminal imaging procedure, euthanize animals according to approved protocol (see Shomer et al. [55])
4.6. Reconstruct the SPECT- or PET-CT images using a 3D fully iterative Monte Carlo-based algorithm
4.7. Ensure that images are calibrated to the injected radioactivity and corrected for tissue attenuation, dead time and radioisotope decay (Note 27^27^)
4.8. Save the data in the standard exchange format for medical images “DICOM” (Digital Imaging Communication in Medicine) and load them in the selected image analysis software (Note 28^28^)
4.9. Before proceeding with image-based analysis, ensure that the CT and SPECT or PET are correctly co-registered
4.10. Delineate regions of interest (ROIs) and quantify corresponding radioactive signal (Note 29^29^ and Note 30^30^)
4.11. Non normalized results can be expressed as percent injected dose (%ID). Normalized results can be expressed as (i) percent injected dose per volume (%ID/mL), when considering the individual organs/tissues volumes, or (ii) standardized uptake value (SUV), with the latter accounting for the average radioactivity across the whole animal (Note 31^31^).
4.12. Express image data as either %ID (Equation 7) or %ID/mL (Equation 8). To best convey the quantitative information and to greatly facilitate the comparison of images across different studies, it is important to apply the same intensity scale (Note 32^32^). The corresponding intensity scale bar should be always present for each figure. For more guidelines on radionuclide-based image analysis, refer to Weber et al. [58] For those with little to no experience in nuclear-based image analysis, refer to a recently published protocol from Cawthorne et al. [59]

5. *Ex vivo biodistribution:*
  5.1. Following to step 4.5, measure the whole animal radioactivity and note the value and time of measurement
  5.2. Proceed with animal dissection and harvest relevant organs and tissues
  5.3. Measure the radioactivity in the tail and in the remaining carcass post dissection (remember to take note of values and time of measurement) (Note 31)
  5.4. Selected organs and tissues are collected in pre-weighted tubes and weighted
  5.5. Prepare radioactive calibration standards with a known activity to simplify decay correction:
    5.5.1. On the day of the experiment, prepare standards from the same stock solution used for radiotracer injection
    5.5.2. Prepare duplicate samples in 1.5 mL Eppendorf tubes
    5.5.3. Each duplicate will be read at the gamma counter before and after your experimental samples using the same settings (*i.e.,* exposure time and energy window)
    5.5.4. At the end of the measurements, a calibration curve (activity *vs* counts per second) can be generated by linear regression
  5.6. Measure radioactivity in all selected organs and tissues alongside with radioactive calibration standards using an automated gamma counter (note the time of measurement) (Note 33^33^)
  5.7. Express values as %ID (Equation 7), %ID/g (Equation 8 Equation 8) or SUV (Equation 9), where the latter are calculated using the following formulas:
    7 $$\% ID=\left(\frac{decay\ corrected\ activity\ (organ)}{decay\ corrected\ injected\ activity\ \left( whole- body\right)}\right)x\ 100$$
    8 $$\% ID/g=\left(\frac{decay\ corrected\ activity\ (organ)/ decay\ corrected\ injected\ activity\ \left( whole- body\right)}{mass(organ)}\right)x\ 100$$
    9 $$SUV=\left(\frac{activity\ (organ)/ mass(organ)}{activity\ \left( whole- body\right)/ mass\left( whole- body\right)}\right)$$
  5.8. After gamma counting, any radioactive sample not needed for further downstream validation can be disposed according to institutional radiation safety and waste management guidelines

Additional downstream analyses include (i) autoradiography, (ii) tissue histology (the latter preceded by organ/tissue embedding (sample preparation may differ depending on embedding procedure selected), (iii) tissue homogenization followed by staining for immune cell markers and flow cytometry.

## Conclusions

When rendering immune cell therapeutics *in vivo* traceable is of interest, one should familiarize with concepts such as tracking time, tracking intervals, sensitivity in the detection, probe, and reporter of choice. We recommend you reading two recent publications discussing each of these aspects and subsequent repercussions on successful *in vitro* tracking of immune cells [5, 53]. The same reviews discuss the pros and cons of both direct and indirect labeling approaches and will help you decide what is best for your experimental needs. How a certain radiotracer distributes in a living animal, its metabolism and clearance route are essential pre-requisites for designing your experiment and the correct interpretation of data. For example, ^89^Zr-based radiopharmaceuticals distribute primarily in the liver. Upon cell death, or as a result of demetallation, free ^89^Zr will be largely accumulating in bones, joints, and marrow because of its high affinity for electronegative donor atoms (*i.e.*, oxygen and phosphorus) in the hydroxyapatite of the bone matrix [60]. While free ^89^Zr homing to the bone and ^89^Zr-labeled cells homing to the bone marrow can be reliably differentiated in humans (due to the large size of the bone marrow cavity), it is not possible to do so in a preclinical setting. However, when tracking immunotherapeutics in living animals using ^89^Zr, one could exploit a chelating agent also used in clinical practice, deferoxamine, to capture the free ^89^Zr and excrete it through the renal route [30]. As the above shows, current literature provides numerous examples of immune cell tracking and troubleshooting tips, some of which were also incorporated in this protocol. However, sometimes experiments will require further optimization for both direct and indirect approaches as many factors can influence labeling efficiency, including the radiotracer of choice, cell type, cell size, buffer, temperature, potential risks of radiotoxicity and associated DNA damage.

Mechanisms by which radioactive probes can enter a cell can also differ. When directly labeling immune cell therapies, the probe can be internalized (i) through small molecules transporters, or (ii) can passively diffuse through the lipophilic cell membrane; whereas, when a reporter gene is employed, this can be (i) a transporter, in which case the probe is internalized, (ii) a receptor, thus irreversibly binding to the probe, or (iii) an enzyme, which then modifies the probe structure, resulting in the entrapment and intracellular accumulation of the probe.

We invite the reader to consult available protocols tailored to specific immune cell types (*e.g.*, T cells, Tregs), radioactive probes (*e.g.*, ^89^Zr-oxine [24], [^18^F]BF_4-_ [47, 48]) and/or reporter genes (*e.g.*, NIS) [47, 49, 50].

**Fig. 3:**
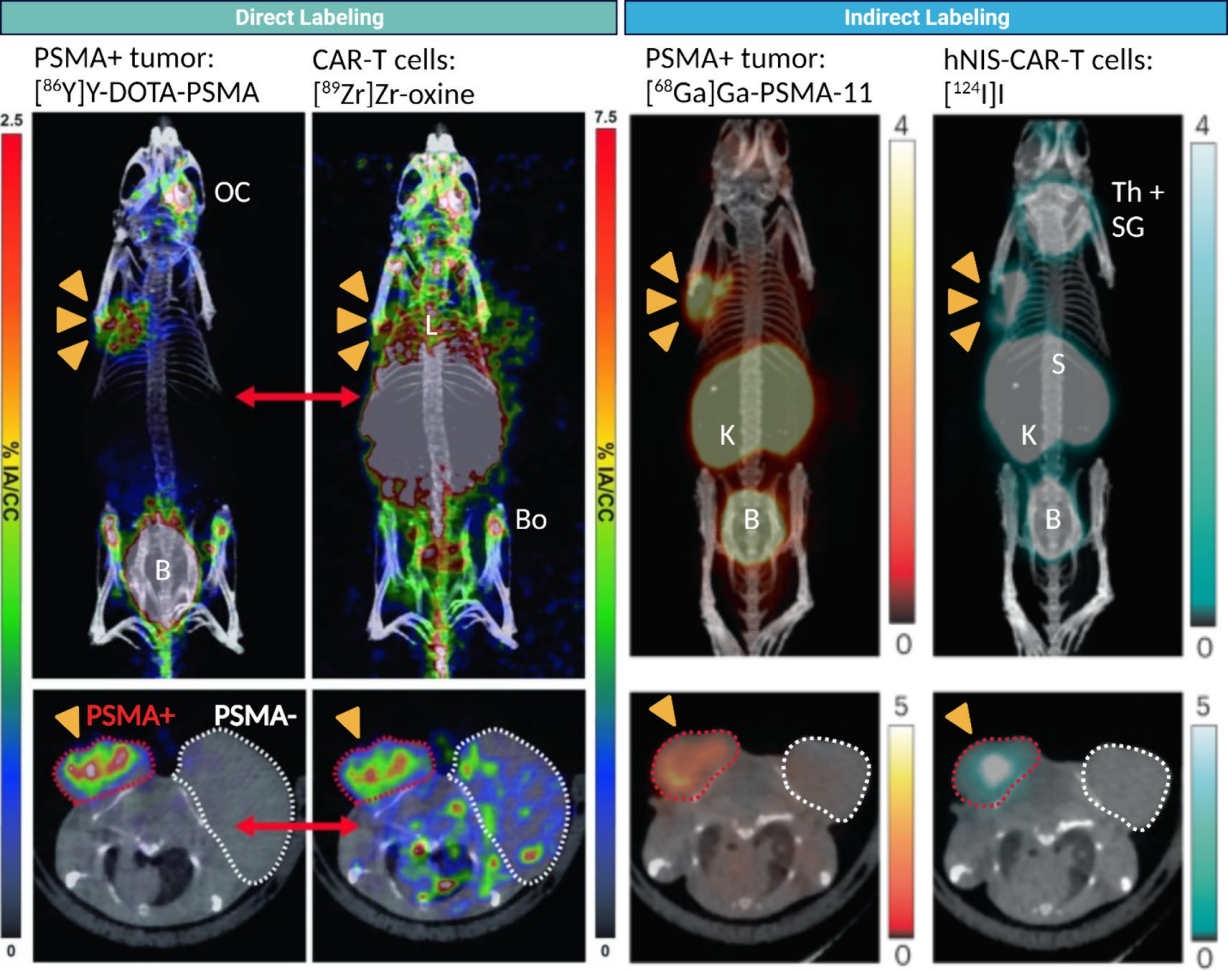
Multiplexed PET-CT imaging of tumor and PSMA-specific CAR-T cell therapy in a preclinical model of prostate cancer. | 3 x 10^6^ PSMA-positive and PSMA-negative tumors were implanted in NOD SCID gamma males (6-8 weeks old). (Right) 5 x 10^6 89^Zr-oxine labelled PSMA-targeting CAR-T cells were systemically infused 25 days post tumor establishment. Alongside ^89^Zr-oxine to image CAR-T cells, an [^86^Y]Y-DOTA-PSMA tracer, retro-orbitally injected, was used to simultaneously image the PSMA-positive tumor using a newly described PET image reconstruction method. [^86^Y]Y-DOTA-PSMA uptake was also seen in the ocular cavity (OC) and bladder (B), the latter as a result of tracer renal excretion. ^89^Zr-oxine signal was also observed in liver (Li) and bones (Bo). (Left) 25 days post tumor establishment, a single dose of 5 x 10^6^ PSMA-targeting CAR-T cells engineered to express hNIS reporter gene were systemically administered. 7 days later, animals were subjected to intravenous injection of ~10.4 MBq [~280 μCi] of [^124^I]I for the NIS-afforded CAR-T imaging, followed one hour later by the infusion of ~13.7 MBq [~370 μCi] of [^68^Ga]Ga-PSMA-11 to identify the PSMA-positive tumors. Isotope separation reveals the anti-PSMA CAR-T successful targeting and homing to the PSMA-positive tumor, as well as the different intratumoral distributions of the two tracers. Reported is also expected uptake in endogenous NIS-expressing tissues: thyroid and salivary glands (Th+SG), stomach (S). [^124^I]I excretion followed the renal route with subsequent uptake in both kidneys (K) and bladder (B). Maximum Intensity Projections (MIPs) and axial slices are shown. Figure adapted form Pratt et al. [9] with permission from publisher

*In vivo* cell tracking is a constantly growing and multidisciplinary field, fueled by the recent advancements in imaging technology. The development of a total-body PET, with its unprecedented sensitivity and resolution [61], permits to extend the *in vivo* tracking time of directly labeled cell-based immunotherapies. Multiplexing capabilities can now be achieved not only via multi-modal imaging (*e.g.*, PET-CT or PET-MRI) [62–64], but also by multiplexed PET [9], with the imaging and quantification of two PET tracers within the same PET acquisition, whereby allowing the simultaneous and independent visualization and targeting of cellular immunotherapies to the tumor using either direct (Fig. 3/Left) or indirect (right) labeling approaches. This is rendered possible by separating and reconstructing double coincidences from recovered triple coincidences via an additional reconstruction algorithm [65].

## Materials

1. *Direct (ex vivo) labeling:*
  1.1. Incubator set at 37° C and 5% CO_2_
  1.2. Agitating rack set at 350 rpm
  1.3. Ice-cold Phosphate Buffered Saline (PBS) not containing Ca^2+^/Mg^2-^, pH 7.4, sterile-filtered
  1.4. Automated Cell Counter or hematocytometer
  1.5. 1.5 mL Eppendorf tubes pre-labeled “supernatant”, “wash 1”, “wash 2” and “cells”
  1.6. Benchtop centrifuge
2. *Indirect (reporter gene-based) labeling:*
  2.1. Viral particle generation
    2.1.1. Incubator set at 37° C and 5% CO_2_
    2.1.2. Viral producing cells (from ATCC)
    2.1.3. Growth medium (varying depending on immune cell type)
    2.1.4. Tissue culture treated 6-well or 10 cm plates
    2.1.5. Transfection reagent (*e.g.*, X-tremeGENE™, Lipofectamine™, Polyethyleneimine (PEI))
    2.1.6. Disinfectant reagent (*e.g.*, Virkon, bleach) for decontaminating the area in contact with viral particles
  2.2. Cellular immunotherapies transduction
    2.2.1. RetroNectin or a similar product (*e.g.*, Poly-L-Lysin)
    2.2.2. Incubator set at 37° C and 5% CO_2_
    2.2.3. Fridge or cold room set at 4° C
    2.2.4. Phosphate Buffered Saline (PBS) not containing Ca^2+^/Mg^2-^, pH 7.4, sterile-filtered
    2.2.5. Phosphate Buffered Saline (PBS) not containing Ca^2+^/Mg^2-^, supplemented with 10% Fetal Calf Serum (FCS), sterile-filtered
    2.2.6. Growth medium (varying depending on immune cell type)
    2.2.7. Automated Cell Counter or hematocytometer
    2.2.8. Benchtop centrifuge set at 100 x *g* and 4 °C
    2.2.9. SFCA Syringe filters 0.45 μm
    2.2.10. 10 mL sterile syringes (disposable, non-sterile)
    2.2.11. 15 mL Falcon tubes
    2.2.12. Non tissue culture treated 6-well plates
    2.2.13. Interleukin-2 (IL-2)
    2.2.14. Interleukin-15 (IL-15)
    2.2.15. Disinfectant reagent (*e.g.*, Virkon, bleach) for decontaminating the area in contact with viral particles
  2.3. *In vitro* characterization of transduced cellular immunotherapies
    2.3.1. Flow cytometer (*e.g.*, BD Fortessa, analyzer) equipped with analysis software, 6-laser system (375/405/488/561/633)
    2.3.2. FACS sorter (*e.g.*, BD FACSAria) equipped with analysis software, 6-laser system (375/405/488/561/633) – use a 70 μm nozzle under 20 psi flow pressure
    2.3.3. For constructs already including a fluorescence marker, this can be used for flow cytometric analysis without further antibody staining, otherwise an antibody binding to a known expressed target and conjugated to a fluorophore is required
    2.3.4. for the radioactive uptake assay, please refer to material in 1.
    2.3.5. For localization studies, use a confocal fluorescence microscope equipped with appropriate lasers and filters for the desired fluorophore(s), an image acquisition and analysis software. Antibody products and their concentrations vary in a case-by-case situation. Use 4% (w/v) paraformaldehyde solution for cells fixation and Hoeachst 33342 solution for nuclei staining. Round cover glasses (22x50 mm) and mounting media (*e.g.*, Mowiol 4-88)
3. *In vivo tracking of cell-based immunotherapies in relevant preclinical models*
  3.1. Benchtop centrifuge
  3.2. 1.5 mL Eppendorf tubes
  3.3. Ice bucket for cooling cells prior to administration
  3.4. Anesthetic (*e.g.*, isofluorane) for inhalation
  3.5. Syringes 0.3 mL U-100 insulin (29 gouge preferred, 0.33 x 12.7 mm; sterile, single-use
  3.6. Rodent anesthesia induction chamber equipped with three-way valves, tube mount connector for inlet, PVC tubing for gas inlet, and 22 mm scavenging tube
  3.7. Rodent anesthesia system, including animal face-mask suitably sized for either mouse or rat and isofluorane vaporizer
  3.8. Veterinary scavenging unit – 240V with automatic weighing mechanism, variable speed control and automatic temperature compensation
  3.9. Preclinical multi-modal SPECT- and PET-CT scanner (microSPECT and PET-CT)
4. *Nuclear imaging by microSPECT-CT and microPET-CT and data analysis:*
  4.1. For *in vivo* imaging, please refer to material in *2.*
  4.2. For *in vivo* data reconstruction, an image analysis software based on full 3D iterative algorithm is required
  4.3. For *in vivo* data analysis, please refer to Note 28 for a full list of available analysis software packages (free or commercially available). Commercially available licenses can often be purchased with the scanner. All are able to co-register SPECT or PET with CT anatomical reference
5. *Ex Vivo Biodistribution:*
  5.1. Glass tubes
  5.2. Automated Gamma Counter with selected energy window (keV; varying depending on radiotracer)

## Footnotes

1 Note 1. As successful radiolabeling with radioactive probes of most immune cells could be negatively affected by their cell culture medium [30], we suggest avoiding it.

2 Note 2. Importantly, some immune cells, such as Tregs, may prefer HBSS (Hank’s Balanced Salt Solution) instead of PBS. We therefore recommend performing a test radiolabeling in different buffers as the optimal conditions may vary depending on cell type [31].

3 Note 3. To compensate for signal loss due to efflux, signal dilution (particularly in highly proliferative cells such as immune cells) and decay, we recommended to use long lived radioisotopes, such as ^89^Zr, ^64^Cu and ^124^I. For a full list of radioactive isotopes and probes used for cell labeling and their respective half-lives, refer to Volpe et al. [22]

4 Note 4. Add required controls: *i.e.*, non-treated (non-radioactive) cells.

5 Note 5. for *in vivo* therapeutic studies number of cells can be scaled up accordingly.

6 Note 6. The incubation temperature may differ depending on the selected radiopharmaceutical probe. Example: ^89^Zr-oxine radiolabeling efficiency is higher when incubation is performed at 4 degrees Celsius as compared to 37 degrees Celsius [30].

7 Note 7. As the assay requires measuring multiple samples, we recommend using a fully automated gamma-counter with applied automated decay correction.

8 Note 8. Viral constructs are typically of retro-, adeno-, and lenti-viral origin and are ectopically. introduced into cells by random integration into the host genome through stable transduction. Alternative methods are based on episomal plasmids (transposons) [32, 33] or gene editing [34–36].

9 Note 9. The “regulatory complex” is the promoter, which can either be classified as: (i) “constitutive”, when it is always “on”, thus providing a continuous transcription, hence better suited for the *in vivo* monitoring of immune cell viability, targeting and persistence; or (ii) “inducible”, leading to a controlled expression of the transgene upon a specific endogenous signal, thus better suited for the *in vivo* monitoring of cell activation and function [2, 37, 38].

10 Note 10. Amphotropic viral-producing cells are required when infecting mammalian cells, including human-derived immune cells.

11 Note 11. Remember that cells in the exponential growth phase produce viral particles with higher titer. Avoid reaching 100% confluence, as this will reduce transfection efficiency and viral titer over time.

12 Note 12. This step is required to remove cells or debris present in the supernatant.

13 Note 13. IL-2 is used during transduction and is replaced 48h post transduction by IL-15 (10 ng/mL) [43] and/pr other cytokines depending on the cell type (*i.e.*, 10 ng/mL TNF-α for dendritic cells) [44]. We refer the reader to Pilipow *et al.* [45] for a description of IL-15 role in both innate and adaptive immune cells. A strategy based on IL-4Rα (an IL-2/IL-15Rβ chimera), allowing *in vitro* selective expansion of CAR-T cells using IL-4 instead of IL-2 or IL-15 was previously described [46] and was used in combination with the sodium iodide symporter (NIS)-RFP dual reporter to generate *in vivo* traceable CAR-T cells [47].

14 Note 14. Immune cells often show a clustering behaviour promoting interactions and information exchange. We therefore recommend adding an additional step before collecting them. Gently pipette them using a serological pipette to obtain a more homogeneous cell suspension ready for counting.

15 Note 15. Supernatants containing two distinct vectors from different simultaneous transfections can be added together for a final volume of maximum 6 mL per well.

16 Note 16. Avoid high speed (*e.g.*, 1000 x *g*) as this could negatively impact immune cells viability.

17 Note 17. Multiple rounds of transduction are recommended to increase transduction efficiency by using fresh viral particles.

18 Note 18. “Control samples” will show the extent of basal uptake in the parental cells.

19 Note 19. Multiply 100 μL aliquots in steps 2.3.3.5 and 2.3.3.6 with a factor of 10 as only 1/10^th^ of the total volume was collected for gamma counting. Calculate averages and standard deviations from triplicate samples.

20 Note 20. Maximum injectable volume corresponds to 10% of a mouse total blood volume (*e.g.*, if the mouse weights 25g, the blood content is 2mL). Desired concentration for injection may vary.

21 Note 21. Ensure ophthalmic ointment is applied to the animals to prevent eye dryness while under anesthesia.

22 Note 22. Route of injection may change depending on experimental design (intravenous, intratumoral, intraperitoneal etc.).

23 Note 23. All reporter genes available, their differing detection sensitivities, as well as paired radionuclide imaging agents, are in Volpe *et al.* [22, 53] If a host reporter is chosen, the researcher should be aware of the potential unfavorable contrast. Therefore, reporters within this class (*e.g.*, human prostate specific membrane antigen: hPSMA; human norepinephrine transporter: hNET; human sodium iodide symporter: hNIS), need to be strategically selected based on the expected distribution of the cellular immunotherapy and the anatomical localization of the target tissue.

24 Note 24. Consecutive imaging is not affected by the previous ones as long as radioactive probe decay is allowed between sessions (*e.g.*, consecutive NIS-based SPECT imaging with [^99m^Tc]TcO_4_^-^ (half-life 6.02h) is possible when scans are 24h apart). [54]

25 Note 25. If the goal is to image cell immunotherapy dynamics (including initial *in vivo* distribution and targeting), a so-called “dynamic” PET imaging should be performed. The latter implies the following steps: (i) inject cells intravenously using a catheter or as a direct injection while animal is placed on the scanner bed and under anesthesia; (ii) acquire list-mode PET data while infusing the cells and continue up to 1-2h post injection. Otherwise, a static (single time frame) PET imaging can be performed at the desired time points to image the long-term distribution of cells. If unsure about the initial distribution of the cells of interest, we suggest starting with a dynamic scan followed by a static scan at a later point.

26 Note 26. The number of imaging sessions that can be performed is strictly dependent on the labeling strategy selected (refer to discussion).

27 Note 27. Refer to scanner’s manufacturer’s instructions.

28 Note 28. Several analysis software packages are available for free, including AMIDE, 3D Slicer and the PET/SPECT-CT Viewer plugin for Fiji/ImageJ, while others are only commercially available, including PMOD, OsiriX DICOM Viewer, MIMviewer, VivoQuant, Carimas and Inveon Workplace.

29 Note 29. If an accurate and unbiased differentiation of relevant signal from surrounding tissues is needed, we suggest using either a (i) manual, or (ii) thresholding-based, (iii) stochastic-based, (iv) region-based or (v) multimodality method of segmentation. [56] Otsu thresholding for image segmentation is also available and can be used for the 3D rendering of regions of interest. [57] For accuracy, we also suggest normalizing the radioactivity values or the single organs/tissues to their volume.

30 Note 30. When using indirect, reporter-based labeling approaches, only viable therapeutic cells will display radiotracer uptake. Thus, a more accurate quantification of their retention at the targeted tissues and/or distribution heterogeneity can be performed.

31 Note 31. We recommend excluding the tail from the measurement of total activity per whole animal. In fact, the activity present in the animal tail is likely due to operator mis-injection, hence, it never reached the blood circulation. As such, it did not contribute to the injected dose and can only serve as an indicator of injection quality.

32 Note 32. All available analysis software allow the user to adjust the minimum and maximum pixel values for image displaying and comparison. The scale used indicates the extent of contrast.

33 Note 33. If radioactive counts are too high (as per detector dead time values or because outside the linearity of the radioactive calibration standards), wait one radioactive half-life later for re-counting.
